# Refractive error and biometrics of anterior segment of eyes of healthy young university students in Japan

**DOI:** 10.1038/s41598-019-51920-4

**Published:** 2019-10-25

**Authors:** Kumiko Kato, Mineo Kondo, Maki Takeuchi, Koji Hirano

**Affiliations:** 1 Department of Ophthalmology, Mie University School of Medicine, Tsu, Japan; 2 Department of Ophthalmology, Bantane Hospital, School of Medicine, Fujita Health University, Nagoya, Japan

**Keywords:** Physiology, Visual system

## Abstract

To determine the parameters of the anterior segment of eyes that are significantly associated with the refractive error in healthy young Japanese university students. This was a cross-sectional observational study of 229 healthy Japanese university students (men: women,147:82) whose age ranged between 20 to 29 years. Univariate and multivariate linear regression analyses were performed to identify the factors that were significantly correlated with the refractive error. The independent variables included age, sex, axial length, anterior chamber depth, corneal diameter, curvature of anterior surface of cornea, and central corneal thickness. The mean refractive error (spherical equivalent) was −4.1 ± 2.7 diopters (D) with a range of −12.5 to +0.5 D, and the mean axial length was 25.4 ± 1.3 mm with a range of 22.4 to 29.0 mm. Pearson univariate correlation analysis found that the refractive error was significantly and negatively correlated with the axial length (R = −0.82, *P* < 0.001), deeper anterior chamber (R = −0.30, *P* < 0.001), and larger corneal diameter (R = −0.21, *P* = 0.001). Multiple regression analysis showed that the refractive error was significantly associated with a longer axial length (*P* < 0.001), a deeper anterior chamber (*P* < 0.001), and a flatter corneal curvature (*P* < 0.001).The biometric values of the anterior segment of the eyes should make the eye more hyperopic which would reduce the myopia-inducing lengthening of the axial length.

## Introduction

Axial myopia is associated with a longer axial length (AL)^1^, and the elongation of the AL can lead to structural changes of the retina^2,3^, choroid^4–6^, and sclera^7–9^ mainly in the posterior pole. The changes induce a thinning of the sclera, choroid, and retina, and the thinning can lead to sight-threatening complications including retinal detachments, choroidal neovascularization, glaucoma, and macular atrophy^4–6^.

The stromal and endothelial cells of the cornea are derived from the neural crest cells, and both the sclera and stroma of the cornea consist of type I collagen^10,11^. The biomechanical changes of the sclera in myopic eyes are due to a net loss of the collagen matrix and subsequent thinning of the sclera^12^. It seems reasonable to assume that the changes in the sclera that occur during the elongation of the eye also occur in the cornea.

There have been many studies that focused on the differences in the values of the anterior segment parameters of myopic eyes from that of emmetropic eyes. Significant correlations have been found between the refractive error and the anterior chamber depth (ACD)^13,14^, corneal diameter^15,16^, corneal curvature^17,18^, central corneal thickness^19–22^, and density of the corneal endothelial cells^23,24^. However, the strength of the correlations varied according to the race and age of the individuals. The prevalence of myopia is also known to vary with age^25^, ethnicity^26^, and level of education^27^, and a higher prevalence of myopia has been reported in university students in Japan and other East Asian countries^28^.

There have been many studies performed on the ocular biometry but most of the studies were conducted on older individuals^13,25,29,30^ or on a wide range of ages ranging from adolescence to the elderly^15,19,20,23,31–33^. Considering that aging could be a confounding factor when the ocular biometry is determined, it is reasonable to design a research to minimize the effect of aging. A Medline search did not extract any studies reporting on the correlations between the degree of myopia and the biometrics of the anterior segment of the eye of younger Japanese individuals.

Thus, the purpose of this study was to determine whether there are significant correlations between the refractive error and the AL of the eye and the values of the different parameters of the anterior segment of the eye in healthy young Japanese university students.

## Results

The demographics of the 229 eyes of 229 young subjects are shown in Table 1. There were 147 men (64.2%) and 82 women (35.8%), and the mean age was 23.1 ± 1.7 years with a range of 20 to 29 years. There were 198 of myopia (86.4%) and 57 of high myopia (24.8%). The decimal BCVA was ≥ 1.0 in all subjects, and none of the eyes had complications associated with myopia.

**Table 1:** Difference of demographics between men and women.

Variables	Overall	Men	Women	*P*-value
Age (years)	23.07 ± 1.74	23.21 ± 1.61	22.83 ± 1.96	0.046*
	23 (22~24)	23 (22~24)	23 (22~23)	
Refractive error (diopters)	−4.06 ± 2.70	−4.14 ± 2.71	−3.92 ± 2.70	0.493*
	−4 (−6.00~−2.00)	−4 (−6.06~−2.13)	−4.00 (−5.91~−1.91)	
Axial length (mm)	25.35 ± 1.31	25.63 ± 1.31	24.87 ± 1.17	<0.001^†^
	25.27 (24.41~26.30)	25.67 (24.64~26.50)	24.73 (24.14~25.54)	
Anterior chamber depth (mm)	3.80 ± 0.26	3.85 ± 0.27	3.72 ± 0.24	<0.001^†^
	3.79 (3.63~3.95)	3.86 (3.67~4.01)	3.73 (3.56~3.84)	
Central corneal thickness (µm)	533.79 ± 34.75	534.13 ± 33.79	533.18 ± 36.63	0.844^†^
	533 (510~556)	532 (511~552)	536 (503~557)	
Corneal diameter (mm)	12.05 ± 0.39	12.11 ± 0.37	11.94 ± 1.17	0.002^†^
	12.08 (11.79~12.31)	12.15 (11.86~12.34)	11.93 (11.67~12.36)	
Corneal curvature (mm)	7.83 ± 0.23	7.87 ± 0.22	7.75 ± 0.23	<0.001*
	7.81 (7.69~7.97)	7.84 (7.74~8.02)	7.77 (7.59~7.91)	

Data are expressed as means ± standard deviations in the upper section and median and range in the lower section.

*Difference in medians between men and women Mann-Whitney test.

^†^Difference in means between men and women X^2^ test.

**Figure 1:**
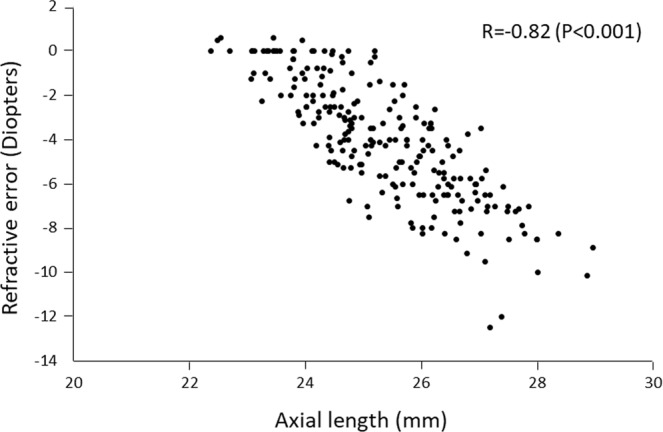
Scatterplots showing the relationship between the refractive error (spherical equivalent) and the axial length. The refractive error is significantly correlated with the axial length (R = −0.82, *P* < 0.001).

**Figure 2:**
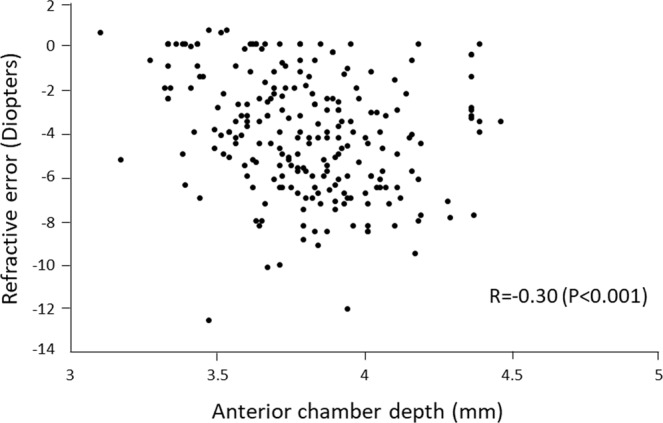
Scatterplots showing the relationship between the refractive error (spherical equivalent) and the anterior chamber depth. The refractive error is significantly correlated with the anterior chamber depth (R = −0.30, *P* < 0.001).

A scatter plot of the refractive errors (ordinate) and AL (abscissa) is showed in Fig. 1, a scatter plot of the refractive errors (ordinate) and ACD (abscissa) in showed in Fig. 2. There were significant differences in the sex, AL, ACD, corneal diameter, and corneal curvature (radius of corneal curvature). The men had significantly longer AL, deeper AC, larger corneal diameter, and flatter cornea (Table 1).

**Figure 3:**
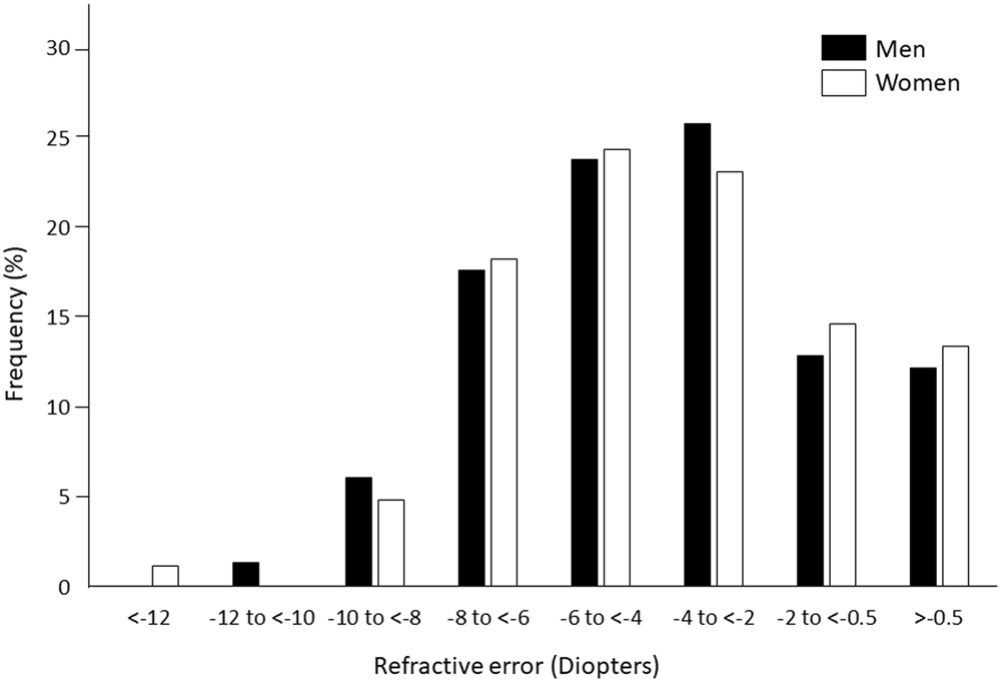
Histogram showing frequency (%) of refractive error in men and women. The difference between men and women was not significant.

The distribution of the refractive errors in men and women is shown in Fig. 3. There was no sex difference in the refractive errors. Pearson univariate correlation analyses showed that the refractive error was significantly and negatively correlated with the AL (R = −0.85 to −0.82, *P* < 0.001) and the ACD (R = −0.37 to −0.26, *P* < 0.001 for all) for all subjects and for the men and women separately (Table 2). There was a weak but significant positive correlation between the refractive error and central corneal thickness for all subjects and for women alone (R = 0.14, *P* < 0.031 for all; and R = 0.30, *P* = 0.006 for women). The corneal diameter was weakly correlated with the refractive error in all subjects and also for men and women separately (R = −0.25 to −0.20, *P* < 0.05). The correlation between the refractive error and the corneal curvature was not significant (*P* > 0.05). The correlations between the refractive error and the corneal endothelial cell density, the standard deviation of corneal endothelial cell density, and the percentage of hexagonal endothelial cells were not significant (data was not shown).

**Table 2:** Univariate correlation between refractive error (spherical equivalent) and biometric parameters of the anterior segment.

	Overall		Men		Women	
	R	*P*-value	R	*P*-value	R	*P*-value
Axial length	−0.82	<0.001	−0.84	<0.001	−0.85	<0.001
Anterior chamber depth	−0.30	<0.001	−0.26	0.001	−0.37	<0.001
Central corneal thickness	0.14	0.031	0.03	0.715	0.30	0.006
Corneal diameter	−0.21	0.001	−0.20	0.017	−0.25	0.023
Corneal curvature	0.00	0.964	0.03	0.743	−0.03	0.805

R; Coefficient of correlation.

Multivariate linear regression analyses were performed with the refractive error as the dependent variable and AL, ACD, corneal diameter, corneal curvature, and central corneal thickness as the independent variables (Table 3). The analyses showed that the AL, corneal curvature, and ACD explained 86–89% of the refractive error variations for all the subjects (R^2^ = 0.86) and the men subjects (R^2^ = 0.89), and the AL and the corneal curvature explained 86% of the refractive error in women (R^2^ = 0.86). The most important contributor to the refractive error was the AL followed by the corneal curvature (Table 3).

**Table 3:** Multivariate analysis of the associations between refractive error (spherical equivalent) and biometric parameters of the anterior segment.

	Variables	Partial regression coefficient	β	P-value	VIF	R^2^ value
Overall	Axial length	−2.244	−1.091	<0.001	1.563	0.864
	Anterior chamber depth	2.134	0.209	<0.001	1.518	
	central corneal thickness	0.001	0.007	0.799	1.070	
	corneal diameter	−0.013	−0.002	0.949	1.505	
	corneal curvature	5.733	0.487	<0.001	1.372	
Men	Axial length	−2.252	−1.092	<0.001	1.478	0.891
	Anterior chamber depth	2.344	0.233	<0.001	1.404	
	central corneal thickness	−0.001	−0.007	0.816	1.042	
	corneal diameter	−0.062	−0.008	0.794	1.333	
	corneal curvature	5.93	0.474	<0.001	1.299	
Women	Axial length	−2.339	−1.014	<0.001	1.452	0.86
	Anterior chamber depth	0.626	0.055	0.377	1.729	
	central corneal thickness	0.001	0.009	0.849	1.164	
	corneal diameter	0.352	0.054	0.351	1.792	
	corneal curvature	4.481	0.384	<0.001	1.435	

^β^Standardized partial regression coefficients.

VIF: Variance inflation factor.

## Discussion

Tideman *et al*. reported that the prevalence of visual impairment rose with increasing axial length and spherical equivalent^34^. Therefore, it is important evaluating structural changes associated with myopia to develop strategies preventing progression of myopia and its complications. The prevalence of myopia (<−0.5 D) in our cohort was 86.4% which was twice that of a population-based study held in Japan in 1997 to 2000 of an elderly population which was 40 to 79 years old^31^. The higher rate of myopia in university students has also been reported in university students in China^35^. In our cohort, the prevalence of high myopia (<−6.0 D) was 24.8% which was higher than the high myopia ratio of 19.5%^35^. In addition, the mean AL in our study group was about 1.3 mm longer than that of the university students in England^28^. Our study group consisted of many myopic subjects, and it was suitable to examine a correlation between the refractive error and ocular biometry.

Our results showed that the AL, the corneal curvature, and the ACD were significantly correlated with the refractive error. The significantly longer AL should lead to higher myopia, but the flatter cornea and deeper anterior chamber would reduce the refractive error (Table 3). A flatter cornea has lower refractive power and the overall refractive power of the eye will be lower, and the light will focus on a point farther from the cornea, i.e., a hyperopic shift. When the anterior chamber gets deeper, the distance between the cornea and crystalline lens increases because the summed power of two lenses is equal to their sums minus the distance between the lenses divided by the index of refraction of the medium. This will also lead to a hyperopic shift. These results indicate that the overall refractive power of the anterior segment of the eye is decreasing, a hyperopic shift, while the AL is increasing, a myopic shift. These compensatory changes lead to an emmetropization of the eye. Our results are in good agreement with the results of earlier studies conducted in other countries that showed that the cornea is flatter in myopic subjects^24,29,30^.

A deeper anterior chamber in myopic subjects has been reported to be present in middle age and elderly individuals in many studies^13,29,32,36,37^. However, the relationship between the central corneal thickness and myopia is contradictory. Some studies reported that the central corneal thickness was not significantly correlated with the myopia^33,36,38,39^, other studies reported that there was a positive correlation between the central corneal thickness and the myopia^19,40^, and other studies reported a negative correlation between them^20,22,24^. Although, studies that reported a significant correlation between the central corneal thickness and refractive error, the correlation coefficients were relatively weak, and the significant correlation was seen in only the simple linear regression analysis. The contribution of the central corneal thickness to the refractive error was not significant in the multiple regression analysis, and we suggest that the correlation between the central corneal thickness and myopia is very weak.

Several population-based studies have reported that men were slightly but statistically significantly more myopic than women^36,41,42^. Sex differences in the corneal curvature, AL, and ACD have also been reported in population-based studies^32,35,36^. In our study, there was no significant difference in the refractive error between men and women but there were significant differences in the AL, ACD, and corneal curvature. Thus, women have slightly shorter AL, shallower ACD, and steeper cornea. Considering that the ACD is an important contributor to the refractive error in multiple regression analysis in men but not in women, anatomical differences in the sexes may exist.

There are some limitations of our study. First, this was a cross-sectional study at one time point, and a prospective longitudinal study is needed to determine the anatomical changes of the eye with the progression of axial myopia. However, our results can be referential data that compared emmetropic and myopic individuals in young Japanese university individuals. Second, our study population was relatively small compared with past studies. Third, we did not collect data on the radius of curvatures and thicknesses of the crystalline lens. In the future, we intend to increase the number of subjects and examine other components of the anterior segment of the eye, e.g., the lens, in young Japanese university students.

The biometric evaluations of the different components of the anterior segment of the eye indicate that the values would make the eyes more hyperopic which would counteract the increase in the axial length which would make the eye more myopic.

## Methods

### Study design

Two hundred and seventy-four healthy volunteers were recruited form the students of the Faculty of Medicine, Mie University School of Medicine. The procedures used in this cross-sectional study were approved by the Institutional Review Board of the Mie University Hospital (No. 3086), and they were performed in accordance with the tenets of The Declaration of Helsinki. The students were informed on the purpose of this study, and a signed written informed consent form was obtained from all before the examinations.

### Subjects

We collected data from 274 of students. The inclusion criteria were: age from 20 to 29 years, absence of any corneal disease that could affect the corneal curvature, such as pterygium, keratoconus, and other corneal degeneration or dystrophy. Eyes with a history of ocular surgery and trauma were excluded. We screened for ocular fundus diseases by optical coherence tomography and fundus examinations. Forty-five students were excluded because of a loss of some of their data. In the end, 229 eyes of 229 normal subjects were studied. The values of only right eye of each subject was used in the statistical analyses.

### Ophthalmological examinations

The ophthalmological examinations were begun at 15:00 h in all participants to minimize the daily fluctuation of the ocular biometrics by the biorhythm, and all of the data were collected in a non-cycloplegic state by well-trained examiners. The best-corrected visual acuity (BCVA) was measured with a Landolt C chart. The refractive error (spherical equivalent) was measured to the closest 0.25 diopter (D). The radius of curvature of the anterior surface of the cornea (corneal curvature) was measured with an auto-refractometer (RC-5000^®^; Tomey, Nagoya, Japan), and the average of the longest and the shortest radius of curvature of the anterior surface of the cornea was used for the statistical analyses. The axial length (AL) and the anterior chamber depth were measured by an optical biometer (OA 2000^®^; Tomey, Nagoya, Japan). The ACD was measured as the distance from the anterior corneal apex to the anterior apex of the crystalline lens in the images of the optical biometer. The corneal diameter was measured by a corneal topographer (OPD scan II^®^; NIDEK Co., LTD, Tokyo, Japan), the central corneal thickness, corneal endothelial cell density, the standard deviations of corneal endothelial cell density, and percentage of hexagonal endothelial cell were determined by a corneal endothelial cell analyzer (EM-4000^®^; Tomey, Nagoya, Japan).

### Statistical analyses

We analyzed the data for all subjects and also separately for men and women. The significance of the differences in the sex distribution was determined by *t*-tests or Mann-Whitney *U* tests. The coefficients of correlation were calculated to evaluate the significance of the association between the refractive error and the AL, ACD, corneal diameter, central corneal thickness, and corneal curvature. Multiple regression analyses were used to assess the contribution of AL, ACD, central corneal thickness, corneal diameter, and corneal curvature to the refractive error. Prior to these examinations, we confirmed that the data were normally distributed by Shapiro-Wilk test. The results were considered statistically significant when *P* < 0.05. Statistical analyses were performed with a commercial statistical software package (SPSS for Windows, version 22.0, SPSS Inc., Chicago, IL).

The datasets generated during and analyzed during the current study are not publicly available due to our Institutional Review Board regulations but are available from the corresponding author on request.
